# STAND: Ultrasound of Cerebral Blood Flow During First Verticalization in Acute Stroke—A Prospective Case–Control Study

**DOI:** 10.1002/brb3.70901

**Published:** 2025-09-21

**Authors:** Laurine Bedoucha, Claire Gobron, Fabrice Vallee, Etienne Gayat, Peggy Reiner, Candice Sabben, Michael Obadia, Perrine Boursin, Estelle Dubus, Eric Jouvent, Mikael Mazighi, Lucas Di Meglio

**Affiliations:** ^1^ Hôpital Lariboisière AP‐HP Université Paris Cité, FHU NeuroVasc, StrokeLink Paris France; ^2^ Service De Physiologie Clinique‐Explorations Fonctionnelles, Assistance Publique—Hôpitaux de Paris, Hôpital Lariboisière Paris France; ^3^ INSERM U942, Cardiovascular MArkers in Stressed COndiTions (MASCOT) Université De Paris Cité Paris France; ^4^ Hôpital Fondation Adolphe De Rothschild Paris France; ^5^ Institut Universitaire De France Paris France; ^6^ Optimisation Thérapeutique En Neuropsychopharmacologie, Université Paris Cité, Institut Universitaire De France U1144 Institut National De La Santé et De La Recherche Médicale (INSERM) Paris France

**Keywords:** carotid stenosis | Doppler | first verticalization | ischemic stroke

## Abstract

**Background and Purpose:**

The optimal timing for mobilizing patients during the acute phase of ischemic stroke remains unclear. Prior research has produced conflicting results, often neglecting the impact of upstream arterial stenosis on cerebral blood flow. This study aimed to determine whether early transition to a seated position in the acute phase of ischemic stroke influences intracranial hemodynamics, particularly in patients with significant carotid stenosis.

**Methods:**

In a prospective, bi‐centric, 1:1 case–control observational study (NCT04180826), we continuously and non‐invasively monitored cerebral and systemic hemodynamics during the first authorized transition from supine to a sitting position in patients with ischemic stroke of the carotid territory. Cases were defined as those with homolateral carotid stenosis >50% by NASCET criteria. The primary outcome was a >10% reduction in mean flow velocity (MFV) in the homolateral middle cerebral artery (MCA).

**Results:**

Of 42 screened patients, 36 were included (19 controls, 17 cases). A significant (>10%) MFV drop occurred in 9/17 cases (53%) versus 1/19 controls (5%; *p* = 0.012). Notably, cases with an MFV drop showed no compensatory systemic response (no rise in blood pressure or heart rate). Multivariate analysis revealed that a shorter time from stroke onset to sitting (coefficient = −2.793, *p* = 0.016) and being a case (coefficient = −6.283, *p* = 0.004) independently predicted an MFV decrease >10%. Additional factors associated with significant MFV decline in cases included the absence of a blood pressure increase after verticalization, lower hemoglobin (*p* = 0.007), and higher BNP levels (*p* = 0.024).

**Conclusions:**

Early sitting in the acute phase of ischemic stroke is more frequently associated with marked MFV reductions in patients with carotid stenosis, potentially due to impaired systemic hemodynamic adaptation. These findings underscore the importance of individualized mobilization strategies based on vascular and systemic factors.

## Graphical abstract

This prospective bi‐centric case–control study investigated early sitting (<72 h) in patients with acute ischemic stroke. Continuous monitoring showed that patients with carotid stenosis frequently experienced >10% reductions in middle cerebral artery flow velocity during the first transition to sitting, without compensatory systemic blood pressure response. These findings highlight impaired hemodynamic adaptation and support individualized mobilization strategies based on vascular status.

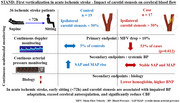

## 1 Introduction

Early mobilization of post‐recanalization stroke patients represents a critical but controversial area of research. Although some studies suggest potential benefits in terms of reduced secondary complications and functional recovery (Govan et al. 2007; Craig et al. 2010), others warn of the risk of worsening cerebral hemodynamic alterations, particularly in the context of the acute phase of stroke (Trial Collaboration Group 2015). These discrepancies highlight the importance of determining the optimal time to initiate mobilization, as well as identifying subgroups of patients likely to benefit from it without compromising their recovery. These aspects are crucial to improve functional prognosis and prevent associated complications.

Cerebral hemodynamics play a central role in these considerations, as they are directly linked to the viability of the ischemic penumbra zone (Goyal et al. 2017). This region of brain tissue is particularly vulnerable, lying between the irreparably damaged infarcted zone and the still‐viable tissue. Its survival depends on the balance between cerebral blood perfusion and local metabolic needs. Even in the event of successful recanalization of the occluded artery, effective perfusion may remain insufficient due to dysfunction of cerebral autoregulation and no‐reflow phenomena (Aries et al. 2010; Sheriff et al. 2023). This makes hemodynamic variations, whether related to systemic factors or changes in body position, particularly critical in the acute phase of stroke.

Body position is known to influence cerebral hemodynamics (Olavarría et al. 2014). The transition from lying to sitting or standing can modify cerebral blood flow, mean arterial pressure, and cerebral blood volume (Low and Singer 2008), parameters crucial to maintaining adequate perfusion in the penumbral zone. However, these interactions between hemodynamics and body position have only been explored outside the acute phase of stroke, even though this period is marked by the most significant pathophysiological challenges.

Extracranial vascular stenoses play a central role in cerebral self‐regulation and the management of vascular resistance. Deweese et al. demonstrated that carotid stenoses can critically alter cerebral perfusion by altering vascular resistance and disrupting cerebral perfusion adaptation (Spencer and Reid 1979; Deweese et al. 1970), particularly in the acute phase, when hemodynamic compensation mechanisms are most strained. However, current data on the interaction between cerebral hemodynamics, vascular stenosis, and variations in body position remain limited. Most studies have been carried out outside the acute phase.

In this context, the objective of this prospective, bi‐centric, case–control study was to determine whether early verticalization in patients with acute ischemic stroke (AIS) is associated with significant cerebral blood flow variations, considering the presence or absence of upstream carotid stenosis or occlusion.

### 1.1 Methods

#### 1.1.1 Study Design and Case–Control Definition

This study was conducted and reported in accordance with the STROBE guidelines for case–control studies to ensure transparency, accuracy, and completeness. This study was a prospective, bi‐centric observational investigation conducted at Fondation Ophtalmologique Adolphe de Rothschild and Hôpital Lariboisière, Paris. Using a 1:1 case–control design, adult patients with AIS or transient ischemic attack (TIA) in the carotid artery territory within 48 h of symptom onset were enrolled between January 2018 and April 2019.

Cases were defined as patients with homolateral carotid stenosis >50%, according to NASCET criteria. Patients over 18 years old, with a Rankin score ≤2 before AIS/TIA and no intracranial stenosis or occlusion, were included.

#### 1.1.2 Standard Protocol Approvals, Registrations, and Patient Consents

The study received ethics committee approval (CPP 2018‐A03454‐51) and was registered on ClinicalTrials.gov (NCT04180826). Emergency inclusion was followed by retrospective patient consent. The objectives, benefits, and risks of participation were explained to patients, and none refused to continue participation.

#### 1.1.3 Hemodynamic Recording and Intervention

Hemodynamic parameters were monitored during the transition from supine to sitting (0°–70°). Intracranial Doppler (ATYS motorized helmet) and systemic measurements, including heart rate and blood pressure, were continuously recorded using the Clearsight system. Baseline data were collected after 5 min in a supine position, followed by verticalization with a 2‐min stabilization at 70°.

#### 1.1.4 Outcomes and Safety

The primary outcome was a >10% decrease in mean flow velocity (MFV) of the homolateral middle cerebral artery (MCA) during verticalization. Secondary outcomes included changes in systemic blood pressure and intracranial Doppler parameters. Safety was evaluated by monitoring adverse events such as dizziness, hypotension, or NIHSS score changes.

More detailed materials are available in the Supporting Information section.

## 2 Results

Between January 2018 and April 2019, 42 patients were screened: 21 cases and 21 controls. Two cases and one control were excluded due to the loss of Doppler signal during verticalization, one case and one control due to the absence of a temporal window, and one case due to the persistence of an intracranial occlusion after acute treatment (Figure S1).

### 2.1 Case–Control Comparison for Primary Endpoint

**FIGURE 1:**
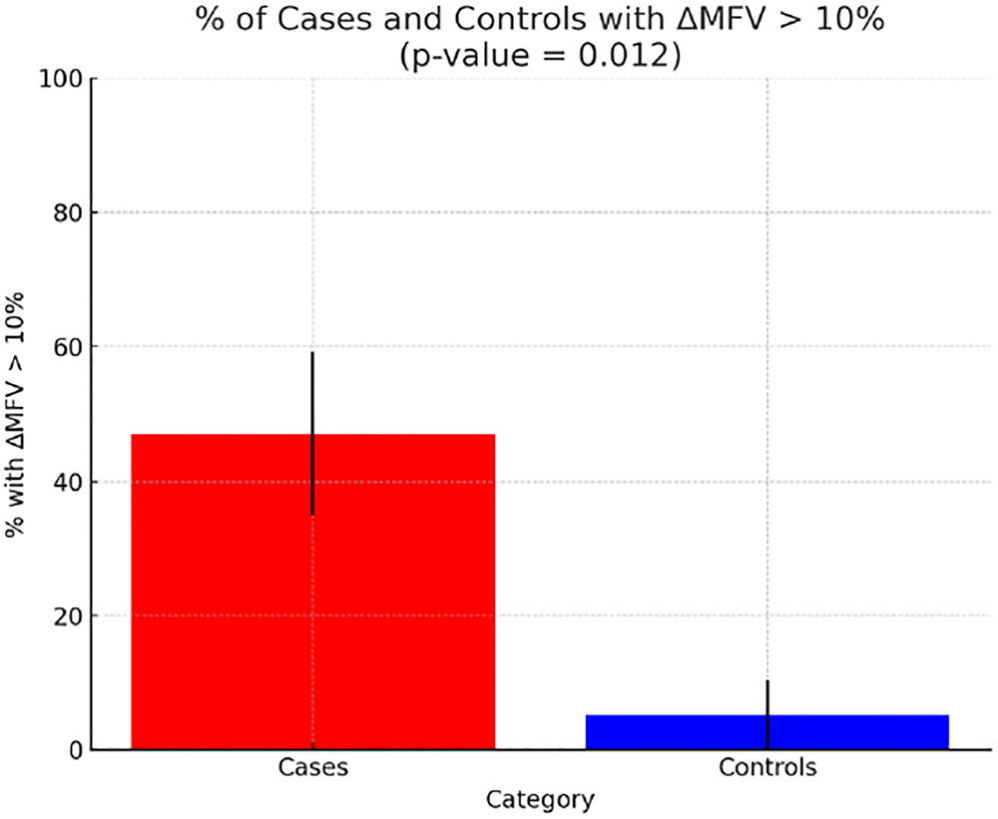
**Primary endpoint: case/control comparison of the percentage of patients with ΔMFV loss >10%**. This bar graph shows the percentage of cases (red) and controls (blue) with a ΔMFV greater than 10% between supine and sitting positions. Error bars represent the standard error. Approximately 47.06% of cases and 5.26% of controls exceed this threshold. The difference is statistically significant (*p* = 0.012).

General characteristics of the population are presented in Table 1. Descriptive statistics and univariate comparisons indicated significant differences between cases and controls for several variables (Table 1). Specifically, nine cases (53%) versus one control (5%) experienced the primary endpoint, a decrease of more than 10% of MFV (*p* = 0.012, Figure 1). The mean delay onset‐to‐first‐verticalization was higher in cases (median 2 IQR [1–3] days) compared to controls (median 1.00 IQR [1–1] days), with a *p* value of 0.0015. Initial intracranial occlusion before treatment was more common in controls (73.6%) compared to cases (29.4%), with a *p* value of 0.020. No significant differences were observed for other clinical or radiological variables. Especially, patients in both groups had similar age, sex, and comorbidities.

**TABLE 1:** Clinical, imaging and biological characteristics of cases and controls.

	Controls *n* = 19	Cases *n* = 17	*p* value
**Cardiovascular risk factors**			
Age, years, mean (std)	70 (14)	67 (13)	0.546
Sex, male (%)	11 (58)	14 (82)	0.156
High blood pressure (%)	11 (61)	11 (65)	1.000
Dyslipidemia (%)	6 (33)	8 (47)	0.499
Diabetes mellitus (%)	4 (22)	5 (29)	0.711
Smoker (%)	3 (18)	5 (29)	0.688
**Medical history**			
Atrial fibrillation (%)	4 (22)	2 (12)	0.658
Stroke (%)	5 (28)	5 (29)	1.000
Coronary artery disease (%)	3 (17)	4 (24)	0.690
mRS before stroke, median (IQR)	0 (0–1)	0 (0–1)	1.000
Prior antihypertensive therapy (%)	11 (53)	9 (58)	1.000
**Stroke characteristics and treatment**			
Ischemic stroke vs. TIA (%)	19 (100)	13 (76)	0.086
Baseline NIHSS, median (IQR)	10 (2–18)	6 (1–13)	0.080
**Initial occlusion (%)**	**14 (73)**	**5 (29)**	**0.020**
Intravenous t‐pa only (%)	4 (21)	1 (6)	0.341
Thrombectomy only (%)	5 (26)	2 (12)	0.408
**Onset‐to‐first transition to sitting position, days, median (IQR)**	**1.00 (1–1)**	**2 (1–3)**	**0.001**
**Vascular imaging features**			
Percentage of homolateral stenosis, median (IQR)	15 (12–17.5)	90 (60–100)	NA
Presence of contralateral stenosis >50% (%)	0 (0)	4 (24)	0.086
Com anterior artery present (%)	15 (79)	13 (76)	1.000
Com posterior artery present (%)	9 (47)	4 (24)	0.177
**Biological tests**			
Hemoglobin, median (IQR)	13.30 (12.75–14.50)	13.20 (11.70–14.20)	0.505
Natremia, mean (std)	140.16 (3.13)	139.82 (1.59)	0.694
Urea, median (IQR)	5.60 (4.5–6)	5.15 (4.34–5.73)	0.486
Creatinine, mean (std)	69.81 (27.63)	74.83 (28.95)	0.426
Protein, mean (std)	67.79 (5.23)	66.13 (6.5)	0.416
BNP, median (IQR)	246 (66–953)	132 (90.8–357)	0.503

**FIGURE 2:**
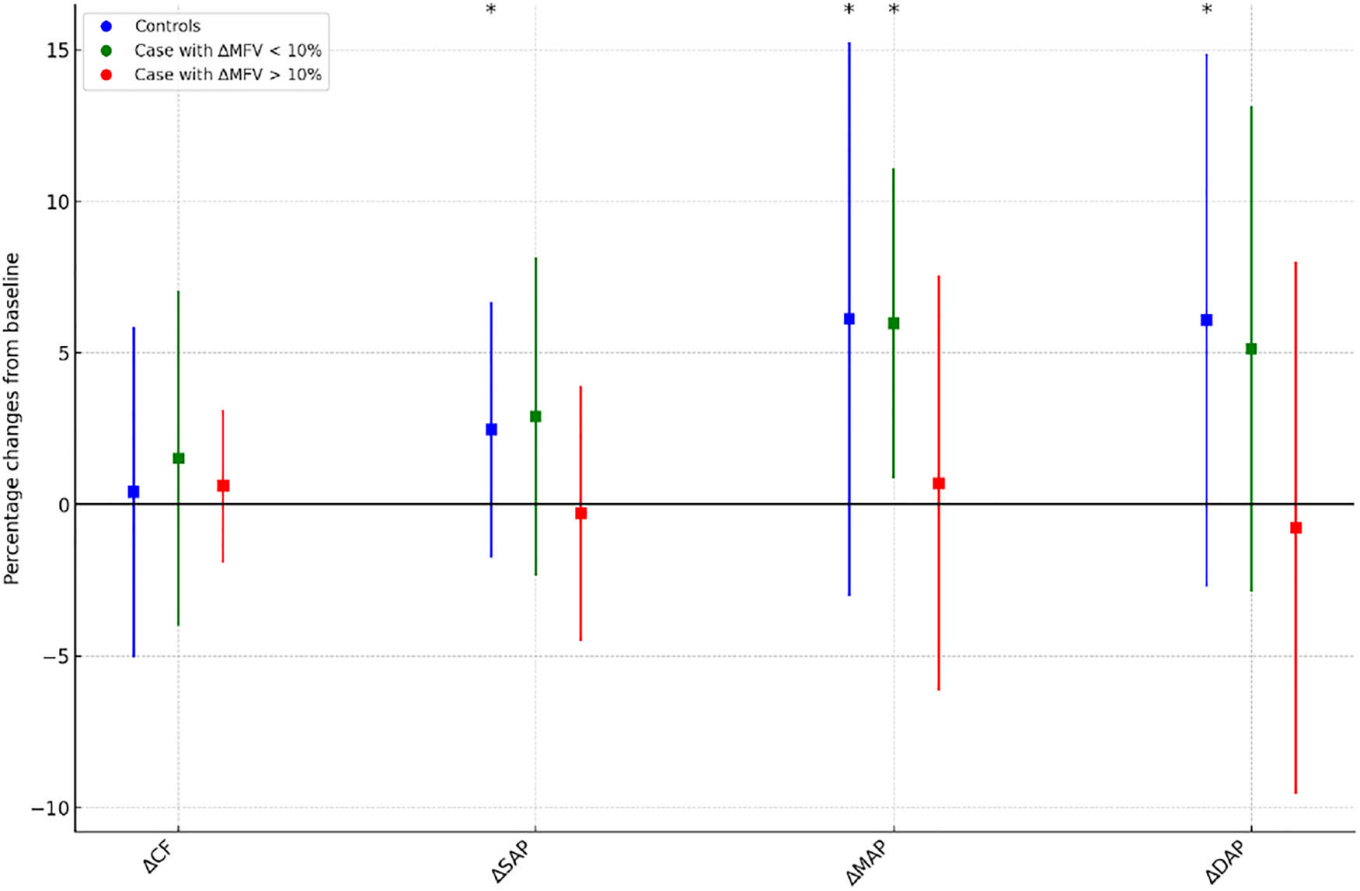
**Percentage changes in systemic hemodynamic parameters from supine to sitting positions**. The graph illustrates the percentage changes in various hemodynamic parameters (cardiac frequency, ∆CF; systolic arterial pressure, ∆SAP; mean arterial pressure, ∆MAP; diastolic arterial pressure, ∆DAP) when transitioning from supine to sitting positions. These changes are depicted for three groups: controls (blue), cases with ∆MFV less than 10% (green), and cases with ∆MFV greater than 10% (red). Error bars represent the standard deviations. Statistically significant differences from zero (*p* < 0.05) are marked with an asterisk. The visualization demonstrates that changes in ∆SAP, ∆DAP, and ∆MAP are significant in the control group, whereas only ∆MAP is significant in the cases with ∆MFV less than 10%. These results suggest that controls exhibit robust increases in arterial pressures upon changing positions, while cases, particularly those with ∆MFV >10%, show a blunted hemodynamic response.

Multivariate logistic regression analysis was conducted to predict the occurrence of the primary endpoint using onset‐to‐first‐verticalization delay, initial intracranial occlusion, and group classification (cases vs. controls) as predictors. The results indicated that onset‐to‐first‐verticalization delay was a significant predictor (coefficient = −2.793, std error = 1.159, *p* = 0.016), as was group classification, where being a control was associated with a lower likelihood of MFV drop >10% (coefficient = −6.283, std error = 2.165, *p* = 0.004). However, initial intracranial occlusion was not a significant predictor in the model (coefficient = 0.419, std error = 1.437, *p* = 0.771). The model had a pseudo *R*‐squared of 0.568 and a log‐likelihood of −8.741, indicating a good fit, which was further supported by the Hosmer–Lemeshow test.

### 2.2 Effect of Verticalization on Hemodynamics

Baseline systemic and intracranial hemodynamic values are presented in Table S1. For intracranial hemodynamics, the only statistical difference observed was a lower pulsatility index in cases (1.28 ± 0.34 vs. 0.97 ± 0.32, *p* = 0.008).

Hemodynamic changes were expressed as a percentage of baseline values. For systemic hemodynamic changes during verticalization (Figure 2), i ncontrols, significant increases were observed in systolic arterial pressure (∆SAP: 8.34% ± 10.39%, *p* = 0.02), mean arterial pressure (∆MAP: 6.12% ± 7.12%, *p* = 0.01), and diastolic arterial pressure (∆DAP: 4.12% ± 5.67%, *p* = 0.01). In contrast, changes in cardiac frequency (∆CF) were not significant (1.15% ± 3.54%, *p* = 0.75). For cases not meeting the primary endpoint (∆MFV drop <10%), there was a significant increase in mean arterial pressure (∆MAP: 6.95% ± 7.89%, *p* = 0.01), but changes in systolic arterial pressure (∆SAP: 5.62% ± 8.23%, *p* = 0.13), diastolic arterial pressure (∆DAP: 4.83% ± 8.23%, *p* = 0.09), and cardiac frequency (∆CF: 1.96% ± 5.23%, *p* = 0.43) were not significant. In cases meeting the primary endpoint (∆MFV drop >10%), no significant changes were observed in any hemodynamic parameters, including cardiac frequency (∆CF: 2.83% ± 7.29%, *p* = 0.51), systolic arterial pressure (∆SAP: −0.87% ± 9.45%, *p* = 0.85), mean arterial pressure (∆MAP: 0.42% ± 6.89%, *p* = 0.78), and diastolic arterial pressure (∆DAP: −1.34% ± 7.87%, *p* = 0.81).

Regarding tolerance, only one case, which had a significant decrease in his MFV, complained of a worsening of an upper limb motor deficit, with an increase of 2 points on the NIHSS, and returned to normal after being laid down. There were no other adverse events.

### 2.3 Other Factors Associated With MFV Decrease in Cases

Cases meeting the primary endpoint (∆MFV drop >10%) (Table S2) had significantly lower hemoglobin levels (12 [11–12] vs. 14 [14–14]g/dL, *p* = 0.007), higher BNP levels (84 [50–116] vs. 322 [209–478]pg/mL, *p* = 0.024), and a shorter onset‐to‐first‐verticalization delay (1.00 [1.00–1.25] vs. 3.00 [2.00–3.00]days, *p* = 0.001) compared to those with cases that did not meet the primary endpoint (∆MFV drop <10%). There were no significant differences observed in age, NIHSS, natremia, urea, creatinine, protein levels, ischemic stroke versus TIA occurrence, sex, stroke occurrence, coronary artery disease, initial occlusion, presence of anterior and posterior communicating arteries, contralateral stenosis >50%, atrial fibrillation, and prior antihypertensive therapy.

## 3 Discussion

This study demonstrates that early transitioning to an upright position in AIS patients and carotid stenosis leads to a significant decrease in ipsilateral MCA MFV compared to those without stenosis. Notably, this reduction in flow velocity appears to stem more from a failure of systemic hemodynamic adaptation than from stenosis severity alone. These findings are clinically relevant and shed new light on optimizing early rehabilitation strategies, particularly for patients with carotid stenosis.

Although previous studies have explored carotid stenosis and its impact on cerebral hemodynamics (Zarrinkoob et al. 2019; Archie and Feldtman 1982; Shakur et al. 2014), the current study is the first to specifically examine its influence during the very early phase of mobilization after AIS. Employing continuous multimodal monitoring—combining transcranial Doppler (TCD), blood pressure measurements, imaging, and biological assessments—strengthens this study by providing a more comprehensive understanding of the complex interactions between systemic and cerebral hemodynamics in the acute phase. This integrated real‐time approach enhances the precision and safety of early mobilization protocols.

These results may also help explain inconsistencies in prior work on very early mobilization (Craig et al. 2010; Lynch et al. 2017; Mariana de Aquino Miranda et al. 2023). Previous trials (Lynch et al. 2017; Mariana de Aquino Miranda et al. 2023) did not adequately account for the presence of carotid stenosis. In this study, patients with carotid stenosis who experienced significant MFV reductions were also those mobilized earlier after stroke onset. This finding aligns with prior studies suggesting that the development of collateral circulation, such as through the circle of Willis, is a time‐dependent process that may not be fully established during early mobilization (Romero et al. 2009). Consequently, these results support the notion that distinct timelines for mobilization could be tailored for patients with and without carotid stenosis to optimize outcomes.

Understanding cerebral hypoperfusion in this context requires considering the interplay between: (i) impaired cerebral autoregulation; (ii) compromised systemic cardiovascular adaptation to posture; and (iii) the direct hemodynamic effects of carotid stenosis.

1. Cerebral autoregulation is frequently impaired in the acute phase of ischemic stroke (Aries et al. 2010), reducing the brain's ability to maintain stable perfusion in response to systemic hemodynamic changes. The plateau of autoregulation, already narrowed in this context, is further challenged by upright positioning, which reduces cerebral perfusion pressure (Garrett et al. 2017). Under normal conditions, this drop is counteracted by a baroreflex‐mediated rise in systemic blood pressure and by cerebral vasodilation, both of which help stabilize cerebral blood flow despite postural stressors (Olavarría et al. 2014; Aries et al. 2013). Anemia adds another layer of complexity: To compensate for reduced oxygen‐carrying capacity, it induces cerebral vasodilation, thereby bringing cerebrovascular reserve closer to its limit (Borgström et al. 1975). Consequently, when both upright posture and anemia are present, the capacity to buffer perfusion changes may be significantly reduced.
2. The transition to an upright position also requires systemic cardiovascular adaptations, including a reflex increase in heart rate, vasoconstriction, and cardiac output, primarily mediated by the baroreflex (Fedorowski et al. 2022). In our cohort, patients with significant MFV drops often failed to exhibit a compensatory rise in systemic blood pressure, suggesting impaired systemic adaptation. Although baroreflex dysfunction related to carotid stenosis (Kitagawa 2010) likely plays a role, other mechanisms may contribute. Stroke‐induced cardiac dysfunction may impair the heart's capacity to increase output (Scheitz et al. 2022), as supported by elevated BNP levels in affected patients. Autonomic dysregulation, common after stroke (Dütsch et al. 2007), may also blunt sympathetic responses. In addition, arterial stiffness—due to aging or chronic hypertension (Saz‐Lara et al. 2023)—may limit vasoconstrictive responses, further hampering postural adaptation.
3. Carotid stenosis itself disrupts cerebral hemodynamics via multiple pathways. The degree of luminal narrowing directly limits blood flow to the ipsilateral hemisphere (Shakur et al. 2014). Moreover, the stenotic segment may impair afferent baroreceptor signaling, weakening the reflex loop that governs systemic pressure regulation (Kitagawa 2010). In these patients, even modest positional challenges may unmask the inability to preserve cerebral perfusion.

Our findings suggest that cerebral hypoperfusion after verticalization in patients with carotid stenosis results from the convergence of these mechanisms: a reduced autoregulatory reserve (exacerbated by anemia), impaired systemic cardiovascular adaptation, and the direct hemodynamic consequences of carotid narrowing. Furthermore, the male predominance in the stenosis group, although not statistically significant, raises the possibility of sex‐related differences in cerebrovascular regulation. A recent study (Mazzucco et al. 2024) has shown that cerebral blood flow velocities vary by sex and are influenced by hemoglobin levels, which modulate intracranial arterial calibers. This is particularly relevant to our findings, as we observed an association between lower hemoglobin and greater MFV drops during mobilization. These mechanisms likely interact, compounding the risk of cerebral hypoperfusion during early mobilization.

These findings underscore the need for individualized mobilization strategies in AIS patients, taking into account carotid status, continuous hemodynamic monitoring, and correction of modifiable factors such as anemia. In current practice, early mobilization protocols are often applied uniformly, without accounting for individual vascular profiles. Our data suggest that in patients with significant carotid stenosis, early upright positioning may lead to impaired cerebral perfusion due to a lack of compensatory systemic adaptation. This supports a more tailored approach to post‐stroke mobilization, where vascular imaging and continuous monitoring could help identify patients at risk of hemodynamic compromise. In such cases, delayed or gradual mobilization, anemia correction (a recent randomized trial suggested that a hemoglobin threshold of 9g/dL instead of 7g/dL improved neurological outcome in patients with acute brain injury [Taccone et al. 2024]), and enhanced systemic monitoring might improve safety and neurological outcomes during early rehabilitation.

However, several limitations must be acknowledged. The sample size, smaller than the initial target of 20 patients per group, reduces statistical power. Another limitation is that the requirement for medical clearance before mobilization may have introduced selection bias by excluding individuals with more severe conditions, potentially reducing the generalizability of our findings. Nonetheless, these safety precautions were considered necessary given the vulnerable patient population. The exclusion of patients with poor temporal windows may have introduced selection bias, limiting generalizability. Although necessary for signal quality, this constraint could be partially addressed in future studies using Doppler contrast agents to improve insonation success rates. The use of TCD as an indirect measure of cerebral blood flow introduces methodological constraints. In particular, TCD does not directly measure expired CO_2_, which can influence intracranial vascular resistance. Despite these drawbacks, TCD remains a practical, non‐invasive, and reliable bedside tool in neurovascular intensive care, providing valuable real‐time data on intracranial hemodynamics (Sarkar et al. 2007). Finally, the >10% MFV drop threshold was defined a priori in our ClinicalTrials.gov–registered protocol, based on available neurocritical care literature (Olavarría et al. 2014) and expert consensus in TCD interpretation. At the time of protocol design, no study had continuously monitored cerebral hemodynamics during early mobilization in AIS, and no validated threshold existed. We therefore adopted a pragmatic cutoff likely to reflect a meaningful change in cerebral blood flow. Although this study was not designed to establish a definitive clinical threshold, it is worth noting that the only patient who experienced transient neurological worsening had an MFV drop >10%, suggesting potential clinical relevance. Future larger studies should investigate whether this threshold is predictive of functional outcomes.

In conclusion, our findings suggest that initiating early verticalization within the first days after an ischemic stroke can lead to a more pronounced decrease in MCA flow velocity among patients with significant carotid stenosis, likely due to inadequate systemic hemodynamic adaptation. Individualized mobilization strategies, along with the identification and correction of contributing factors such as anemia and hypotension before mobilization, may help preserve cerebral perfusion and potentially improve clinical outcomes.

## Author Contributions

**Laurine Bedoucha**: investigation, writing – original draft, methodology, validation, visualization, writing – review and editing, project administration, data curation. **Claire Gobron**: investigation, writing – original draft, writing – review and editing, project administration. **Fabrice Vallee**: writing – original draft, writing – review and editing, project administration, investigation. **Etienne Gayat**: project administration, writing – review and editing, writing – original draft, investigation. **Peggy Reiner**: investigation, writing – original draft, writing – review and editing, project administration. **Candice Sabben**: investigation, writing – original draft, writing – review and editing, project administration. **Michael Obadia**: writing – original draft, writing – review and editing, project administration, investigation. **Perrine Boursin**: writing – original draft, investigation, writing – review and editing, project administration. **Estelle Dubus**: investigation, writing – original draft, writing – review and editing, project administration. **Eric Jouvent**: investigation, writing – original draft, writing – review and editing, project administration. **Mikael Mazighi**: conceptualization, investigation, methodology, validation, visualization, writing – review and editing, software, formal analysis, project administration, data curation, supervision, resources, writing – original draft. **Lucas di Meglio**: conceptualization, investigation, writing – original draft, methodology, validation, visualization, writing – review and editing, software, formal analysis, project administration, data curation, supervision, resources.

## Conflicts of Interest

The authors declare no conflicts of interest

## Peer Review

The peer review history for this article is available at https://publons.com/publon/10.1002/brb3.70901.

## Supporting information




Supplemental Materials

## Data Availability

The author has provided the required Data Availability Statement and, if applicable, included functional and accurate links to said data therein.
