# Supplementary material for: STAND: Ultrasound of Cerebral Blood Flow During First Verticalization in Acute Stroke—A Prospective Case–Control Study
Source: Brain Behav. 2025 Sep 21;15(9):e70901. doi: 10.1002/brb3.70901 (PMC12451070; doi:10.1002/brb3.70901)
Supplement: Supplementary file 1 — Supplemental Materials [file BRB3-15-e70901-s001.docx]

**Supplemental Materials**

**Study design and case control definition**

This is a prospective, bi-centric (Fondation ophtalmologique Adolphe de Rothschild, Paris – Hôpital Lariboisière Hospital, APHP, Paris) observational study of a 1:1 case-control design. Between January 2018 and April 2019, adult patients with an AIS or transitory ischemic attack (TIA) of the carotid artery territory within 48 hours from onset were included in this study.

**Inclusions criteria** were patient over 18 years of age, AIS or TIA with symptom onset less than 48 hours, absence of homolateral or downstream intracranial stenosis or M1 occlusion, verticalization authorized by the referring clinician, Rankin score before AIS/TIA ≤ 2.

**Exclusions criteria** were disrupted vigilance, absence of a temporal window on Doppler ultrasound and lack of medical authorization for the first standing.

**Cases were defined** as patients with homolateral carotid stenosis greater than 50% according to NASCET criteria.

**Standard protocol approvals, registrations, and patient consents**

The study received approval from the ethics committee (CPP 2018-A03454-51) and was registered on ClinicalTrials.gov (<https://clinicaltrials.gov/study/NCT04180826>). The study protocol included emergency inclusion, followed by the retrospective collection of consent for continued participation. No patient refused to continue their participation in the study. The objectives of the trial, its characteristics, as well as the potential benefits and risks, were clearly explained to the patients by the investigator.

**Hemodynamic recording and intervention**

*Intracranial doppler and systemic hemodynamic monitoring*

Intracranial Doppler recordings were continuously obtained using the ATYS® motorized helmet (doppler spectrum) during the initial transition from supine to a sitting position (0–70°) following medical clearance. Simultaneously, systemic hemodynamic parameters—including heart rate, systolic, mean, and diastolic blood pressure—were monitored using the Clearsight® system.

*Baseline hemodynamic assessment in supine position*

Prior to the verticalization procedure, baseline intracranial hemodynamic parameters were collected with the patient in a strict supine position (0–30°) in their hospital bed, according to standard care protocols in the Neurovascular Unit.

To ensure accurate monitoring, the ATYS TCD-X® transcranial Doppler headset was positioned and secured using glasses. Pulsed Doppler signals were verified using transcranial Doppler ultrasound. A digital sensor was attached for continuous blood pressure monitoring, which was integrated with a monitoring system for simultaneous recording of ECG and oxygen saturation. After 5 minutes of stabilization in the supine position, baseline data—including time, middle cerebral artery (MCA) mean velocity, blood pressure, and heart rate—were recorded.

*Verticalization protocol*

The transition from supine (0°) to an upright position (70°) was performed using the bed's electronic controls, ensuring a gradual and continuous adjustment of the tilt angle. The final tilt was confirmed using a bubble level. After a 2-minute stabilization period in the upright position, the same parameters (time, MCA mean velocity, blood pressure, and heart rate) were recorded. Data from transcranial Doppler and systemic hemodynamic monitoring were continuously recorded throughout the procedure.

**Outcomes and safety**

The primary outcome was set *a priori* as the occurrence of a decrease of more than 10% in the averaged mean flow velocity (MFV) in the stroke homolateral middle cerebral artery (MCA) between the supine and sitting positions. The assessment of the primary outcome was conducted within 72 hours after the IS. ACM MFV was calculated over 3 cardiac cycles measured twice: after 5 minutes in a strict supine position, then immediately after 2 minutes at 70° (see verticalization protocol).

Secondary outcomes included changes in various parameters before and after verticalization to 70°. These included transcranial Doppler measurements, such as systolic upstroke time, pulsatility index, resistance index, systolic velocity, and diastolic velocity. Alterations in blood pressure parameters—systolic, mean arterial, and diastolic pressures—were also evaluated. Clinical tolerance to verticalization was assessed by observing changes in the National Institutes of Health Stroke Scale (NIHSS) score, episodes of arterial hypotension, and occurrence of adverse effects such as discomfort or dizziness. Additionally, biological parameters, including levels of creatinine, sodium, proteins, hemoglobin, hematocrit, and brain natriuretic peptide (BNP), were analyzed.

**Data collection**

Collected data included demographic and cardiovascular risk factors, such as age, sex, hypertension, dyslipidemia, diabetes, and smoking history. Medical history, including atrial fibrillation (AF), previous stroke, and ischemic heart disease, was documented alongside treatments at inclusion and during the acute stroke phase, such as beta-blockers, diuretics, and thrombolytic therapy.

Stroke characteristics were recorded, including the initial NIHSS score, the presence and type of arterial occlusion, and other relevant imaging findings. Imaging data at the time of Doppler assessment, such as the degree of stenosis, presence of contralateral stenosis, and communicating arteries, were also documented.

**Sample size**

Sample size calculations were based on detecting a difference in the primary outcome with an effect size that assumed 10% occurrence in the control group and 50% in the case group, a type I error rate of 5%, and a power of 90%. This resulted in a required sample size of 20 subjects per group.

**Statistical analysis**

For the primary analysis, descriptive statistics were calculated for each variable, and comparisons between cases and controls were conducted using appropriate statistical tests. Mann-Whitney U tests were used for ordinal variables, and Chi-Square tests were used for dichotomous variables. Multivariate logistic regression was performed to identify predictors of the primary endpoint. The logistic regression model included significant variables from the univariate analysis.

Model fit was assessed using the Pseudo R-squared and the Hosmer-Lemeshow goodness-of-fit test. Multicollinearity among predictors was checked using the Variance Inflation Factor (VIF), with all VIF values below 10 indicating no significant multicollinearity. Residuals and influential data points were examined using Cook’s distance to ensure model robustness, and no significant outliers or high-leverage points were identified.

Secondary analyses involved evaluating changes in blood pressure parameters before and after verticalization, using paired t-tests or Wilcoxon signed-rank tests as appropriate. Exploratory analyses included subgroup comparisons and additional tests to investigate the effects of different clinical variables on the primary and secondary outcomes.

All statistical analyses were conducted using Python version 3.9 with the Statsmodels library version 0.12.2. Confidence intervals for estimated parameters were calculated and interpreted accordingly. Data were summarized and presented as mean ± standard deviation for normally distributed variables or median [IQR] for non-normally distributed variables. Statistical analyses were conducted under the supervision of a biostatistics expert to ensure methodological rigor and accuracy.

**Supplemental Figure 1: Flowchart**


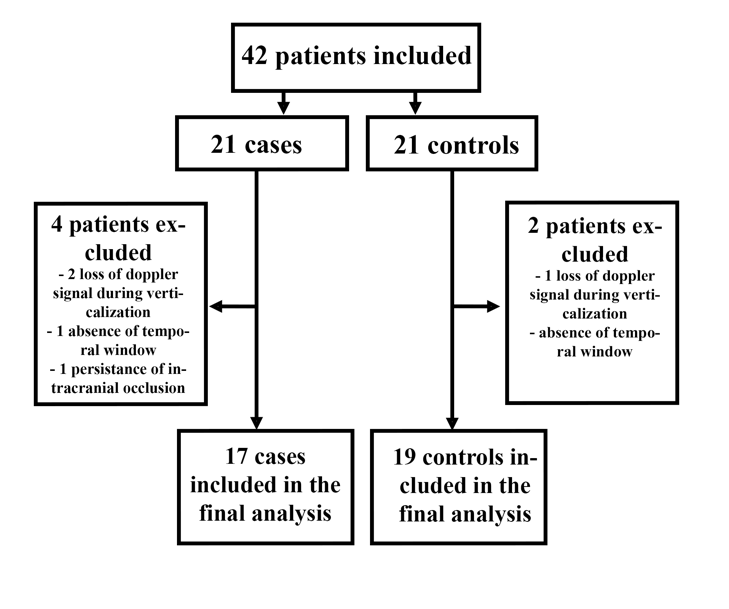


This flowchart illustrates the selection process of the study participants. Initially, 42 patients were included, with 21 cases and 21 controls. Among the cases, 4 patients were excluded due to loss of Doppler signal during verticalization (2 patients), absence of temporal window (1 patient), and persistence of intracranial occlusion (1 patient). Among the controls, 2 patients were excluded due to loss of Doppler signal during verticalization (1 patient) and absence of temporal window (1 patient). Ultimately, 17 cases and 19 controls were included in the final analysis.

**Supplemental Figure 2: Primary endpoint: case/control comparison of the percentage of patient with ΔMFV loss > 10%**


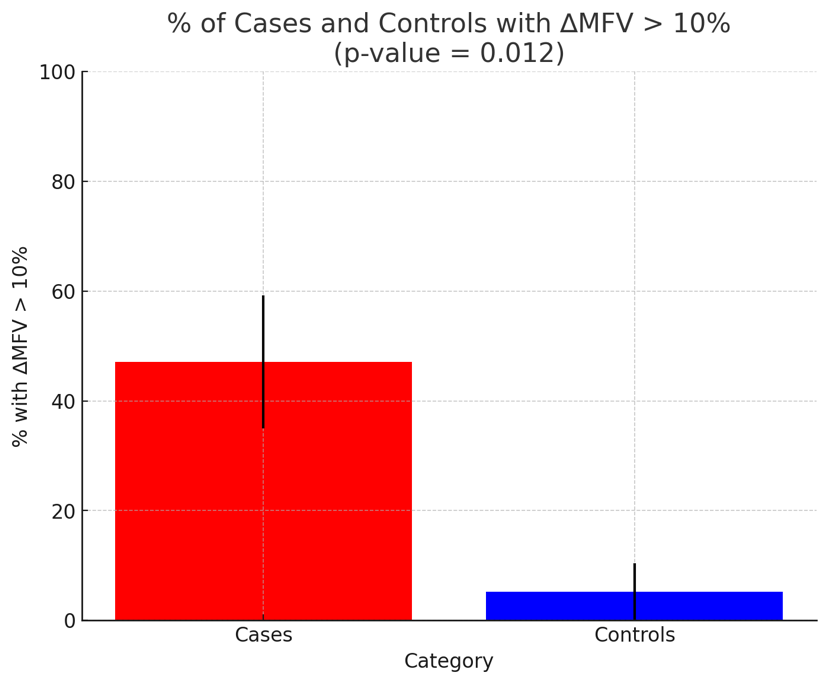


This bar graph shows the percentage of cases (red) and controls (blue) with a ΔMFV greater than 10% between prone and supine positions. Error bars represent the standard error. Approximately 47.06% of cases and 5.26% of controls exceed this threshold. The difference is statistically significant (p = 0.012).

**Supplemental table 1: Baseline systemic and intracranial hemodynamic**

| **SYSTEMIC HEMODYNAMIC** | Control | Case | p-value |
| --- | --- | --- | --- |
| Cardiac frequency_prone, mean (std), bpm | 80.32 (18.03) | 71.47 (12.93) | 0,246829 |
| Cardiac frequency_supine, mean (std), bpm | 80.16 (15.42) | 72.47 (14.92) | 0,124016 |
| Systolic arterial pressure_prone, mean (std), mmHg | 139.26 (20.43) | 134.47 (24.97) | 0,531006 |
| Systolic arterial pressure _supine, mean (std), mmHg | 142.63 (21.02) | 136.41 (25.96) | 0,432911 |
| Mean arterial pressure _prone, mean (std), mmHg | 89.76 (14.50) | 88.53 (17.66) | 0,819438 |
| Mean arterial pressure _supine, mean (std), mmHg | 94.42 (12.83) | 91.82 (19.33) | 0,634485 |
| Diastolic arterial pressure_prone, mean (std), mmHg | 74.16 (14.39) | 73.12 (15.94) | 0,838177 |
| Diastolic arterial pressure_supine, mean (std), mmHg | 78.05 (12.90) | 75.29 (18.76) | 0,607387 |
| **INTRACRANIAL HEMODYNAMIC** | Control | Case | p-value |
| Mean flow velocity_prone, mean (std), cm/s | 28.32 (9.21) | 27.18 (8.89) | 0,750888 |
| Mean flow velocity_supine, mean (std), cm/s | 27.47 (8.31) | 24.82 (7.53) | 0,555994 |
| Systolic velocity_prone, mean (std), cm/s | 86.16 (23.03) | 76.50 (33.26) | 0,314036 |
| Systolic velocity_supine, mean (std), cm/s | 84.58 (22.12) | 71.68 (33.57) | 0,177923 |
| Diastolic velocity_prone, mean (std), cm/s | 27.63 (12.78) | 27.65 (10.74) | 0,811803 |
| Diastolic velocity_supine, mean (std), cm/s | 26.53 (10.31) | 25.38 (7.91) | 0,962034 |
| **Pulsatility index_prone, mean (std)** | **1.28 (0.34)** | **0.97 (0.32)** | **0,008268** |
| **Pulsatility index_supine, mean (std)** | **1.31 (0.33)** | **1.00 (0.32)** | **0,006733** |
| Resistive index_prone, mean (std) | 0.68 (0.08) | 0.67 (0.28) | 0,876735 |
| Resistive index_supine, mean (std) | 0.68 (0.08) | 0.66 (0.24) | 0,715364 |

**Supplemental table 2: Comparison between cases that experienced a decrease in their MFV > 10% upon assuming the seated position and those with stable MFV**

|  | **Cases with ∆MFV < 10%**  **n = 9** | **Cases with ∆MFV > 10%**  **n = 8** | **p-value** |
| --- | --- | --- | --- |
| **CARDIOVASCULAR RISK FACTORS** |  |  |  |
| Age. years, mean (std) | 68 (13) | 66 (15) | 0.785 |
| Sex, male (%) | 8 (89%) | 6 (75%) | 0.910 |
| High blood pressure (%) | 5 (56) | 6 (75) | 0.619 |
| Dyslipidemia (%) | 3 (33) | 5 (63) | 0.346 |
| Diabetes mellitus (%) | 1 (11) | 4 (50) | 0.131 |
| Smoker (%) | 2 (22) | 3 (38) | 0.619 |
| **MEDICAL HISTORY** |  |  |  |
| Atrial fibrillation (%) | 1 (11%) | 1 (13%) | 1 |
| Stroke (%) | 2 (22%) | 3 (38%) | 0.875 |
| Coronary artery disease (%) | 2 (22%) | 2 (25%) | 1 |
| mRS before stroke, median (IQR) | 0.0 (0.0-0.0) | 0.0 (0.0-1.0) | 0.469 |
| Prior antihypertensive therapy (%) | 5 (56%) | 4 (50%) | 1 |
| **STROKE CHARACTERISTICS AND TREATMENT** |  |  |  |
| Ischemic stroke vs TIA (%) | 5 (56) | 8 (100) | 0.113 |
| Baseline NIHSS, median (IQR) | 1 (0-6) | 6 (3-11) | 0.058 |
| Initial occlusion (%) | 1 (11%) | 4 (50%) | 0.221 |
| Intravenous t-pa (%) | 1 (11) | 2 (25) | 0.910 |
| Thrombectomy (%) | 2 (22) | 3 (38) | 0.875 |
| **Onset-to-first transition to sitting position, days, median (IQR)** | **3.00 (2.00-3.00)** | **1.00 (1.00-1.25)** | **0.001** |
| **VASCULAR IMAGING FEATURES** |  |  |  |
| Presence of contralateral stenosis > 50% (%) | 3 (33%) | 1 (13%) | 0.661 |
| Com anterior artery present (%) | 8 (89%) | 5 (63%) | 0.479 |
| Com posterior artery present (%) | 3 (33%) | 1 (13%) | 0.661 |
| **BIOLOGICAL TESTS** |  |  |  |
| **Hemoglobin, median (IQR)** | **14 (14-14)** | **12 (11-12)** | **0.006** |
| Natremia, mean (std) | 140 (139-141) | 140 (2) | 0.906 |
| Urea, median (IQR) | 5 (1) | 5 (2) | 0.752 |
| Creatinine, mean (std) | 80 (15) | 70 (39) | 0.530 |
| Protein, mean (std) | 68 (6) | 65 (7) | 0.434 |
| **BNP, median (IQR)** | **84 (50-116)** | **322 (209-478)** | **0.024** |
